# Supplementary material for: miR-7 Buffers Differentiation in the Developing Drosophila Visual System
Source: Cell Rep. 2017 Aug 8;20(6):1255–61. doi: 10.1016/j.celrep.2017.07.047 (PMC5561169; doi:10.1016/j.celrep.2017.07.047)
Supplement: Document S1. Figures S1 and S2 [file mmc1.pdf]

Cell Reports, Volume *20*

Supplemental Information

miR-7 Buffers Differentiation in the Developing *Drosophila* Visual System

Elizabeth E. Caygill and Andrea H. Brand

Supplementary Figure 1

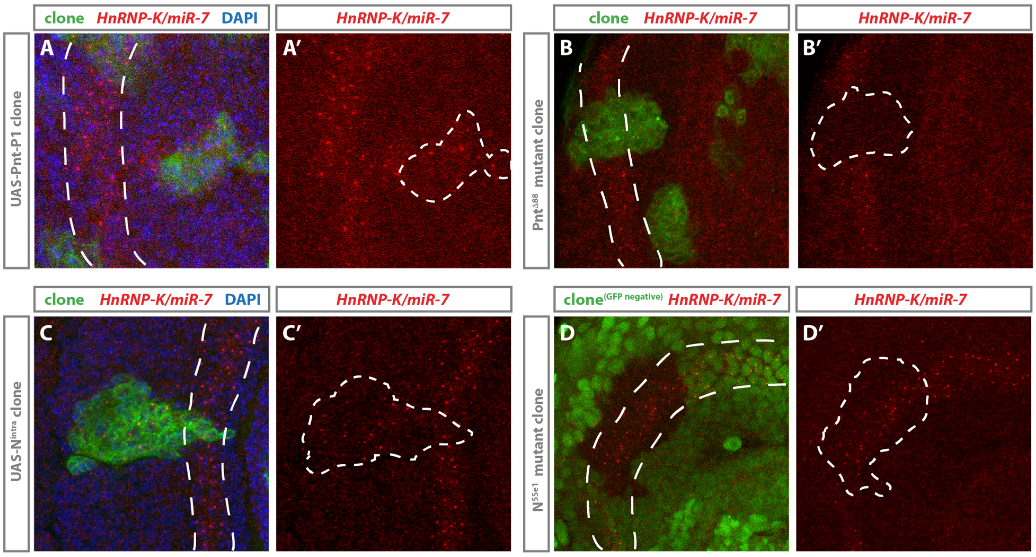

Supplemental Figure 1. hnRNP-K/miR-7 **is regulated by EGF signaling at the transition zone, (Related to Figure 1).**

We tested the ability of *HnRNP-K/miR-7* transcription to respond to alterations in the two major signaling pathways that modulate the transition zone, EGF and Notch. Upregulation of EGF signaling via clonal expression of UAS-Pnt-P1 induces upregulation of *HnRNP-K/miR-7* autonomously and non-autonomously (A). Clonal loss of EGF signaling results in a loss of *HnRNP-K/miR-7* expression (B) suggesting EGF is necessary for expression of *HnRNP-K/miR-7*. Upregulation of Notch signaling via clonal expression of UAS-N^{intra} induces upregulation of *HnRNP-K/miR-7* within the clone (C). However as upregulation of Notch signaling is known to induce EGF activity we tested the effect of loss of Notch signaling and saw that clonal loss of Notch signaling does not result in a loss of *HnRNP-K/miR-7* expression (D) suggesting Notch does not directly regulate expression of *HnRNP-K/miR-7*.

Supplementary Figure 2

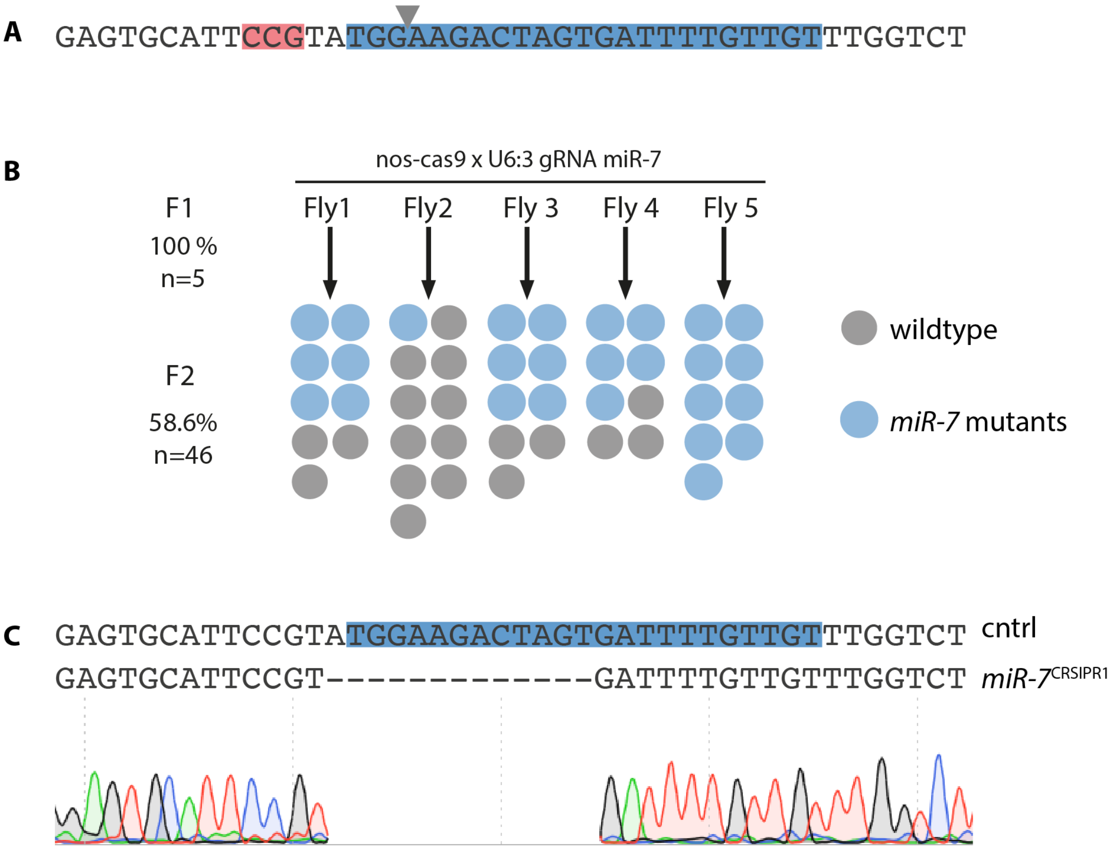

Supplemental Figure 2. **Generation of a *miR-7* CRISPR mutant, (Related to Figure 2).**

(A) The *miR-7* sequence (blue) and surrounding DNA showing the cut site (grey triangle) and the PAM sequence (red) of the gRNA chosen for mutation of *miR-7*.

(B) Efficiency of *miR-7* disruption. nos-cas9 flies were crossed to U6:3 miR-7 gRNA flies. Five F1 male progeny were crossed to balancer lines. 10 F2 male progeny were allowed to fertilize balancer females for 2-3 days then removed from the cross vials and used in diagnostic PCRs. 58.6% (n=46) of F2 progeny tested showed disruption of the *miR-7* locus generating 20 independent alleles.

(C) Sequencing of a *miR-7*^{CRISPR1} homozygous mutant shows a 13bp deletion.
