# Supplementary material for: miR-7 Buffers Differentiation in the Developing Drosophila Visual System
Source: Cell Rep. 2017 Aug 8;20(6):1255–61. doi: 10.1016/j.celrep.2017.07.047 (PMC5561169; doi:10.1016/j.celrep.2017.07.047)
Supplement: Document S2. Article plus Supplemental Information [file mmc2.pdf]

Report

Cell Reports

miR-7 Buffers Differentiation in the Developing *Drosophila* Visual System

Graphical Abstract

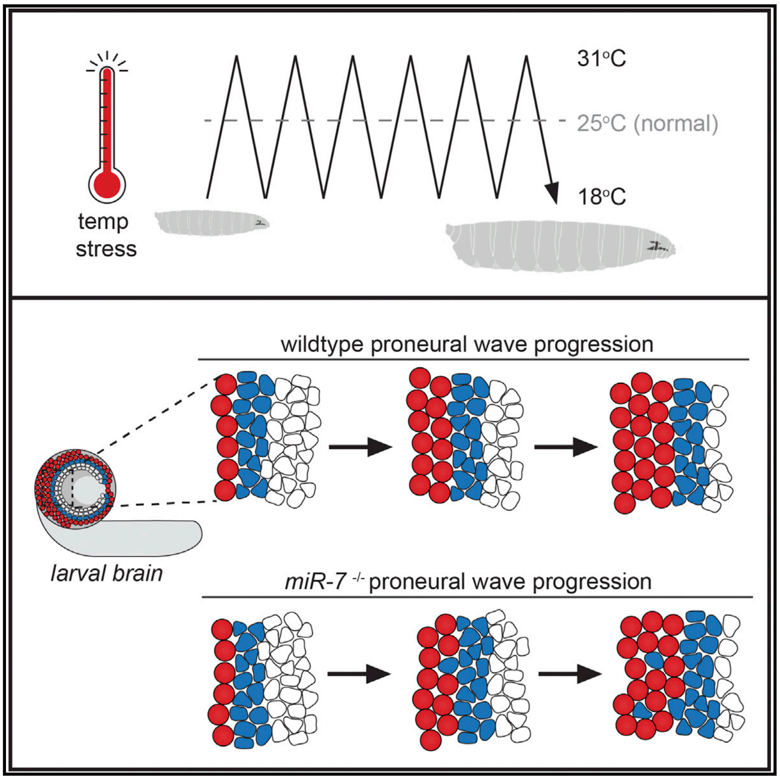

Highlights

- *miR-7* promotes neuroblast formation during optic lobe development
- *miR-7* targets the Notch pathway
- *miR-7* buffers the effects of environmental stress
- Without *miR-7*, timely neuroblast production is disrupted

Authors

Elizabeth E. Caygill, Andrea H. Brand

Correspondence

a.brand@gurdon.cam.ac.uk

In Brief

Caygill and Brand show that *miR-7* buffers optic lobe neural stem cell production to ensure that a precise and stereotypical pattern is maintained, even under conditions of environmental stress. These results echo the role that *miR-7* plays in the eye imaginal disc, emphasizing the importance of robust visual system development.

Caygill & Brand, 2017, Cell Reports *20*, 1255–1261
August 8, 2017 © 2017 The Authors.
http://dx.doi.org/10.1016/j.celrep.2017.07.047

Cell Reports
Report

OPEN ACCESS
CellPress

miR-7 Buffers Differentiation in the Developing *Drosophila* Visual System

Elizabeth E. Caygill[1] and Andrea H. Brand[1,2,*]
[1]The Gurdon Institute and Department of Physiology, Development and Neuroscience, University of Cambridge, Tennis Court Road, Cambridge CB2 1QN, UK
[2]Lead Contact
*Correspondence: a.brand@gurdon.cam.ac.uk
http://dx.doi.org/10.1016/j.celrep.2017.07.047

SUMMARY

The 40,000 neurons of the medulla, the largest visual processing center of the *Drosophila* brain, derive from a sheet of neuroepithelial cells. During larval development, a wave of differentiation sweeps across the neuroepithelium, converting neuroepithelial cells into neuroblasts that sequentially express transcription factors specifying different neuronal cell fates. The switch from neuroepithelial cells to neuroblasts is controlled by a complex gene regulatory network and is marked by the expression of the proneural gene *l'sc*. We discovered that microRNA *miR-7* is expressed at the transition between neuroepithelial cells and neuroblasts. We showed that *miR-7* promotes neuroepithelial cell-to-neuroblast transition by targeting downstream Notch effectors to limit Notch signaling. *miR-7* acts as a buffer to ensure that a precise and stereotypical pattern of transition is maintained, even under conditions of environmental stress, echoing the role that *miR-7* plays in the eye imaginal disc. This common mechanism reflects the importance of robust visual system development.

INTRODUCTION

Drosophila vision requires the accurate specification of over 80 different types of optic lobe neurons and the establishment of precise visual circuits between the neurons of the optic lobe and the photoreceptors of the eye. The medulla is the largest visual ganglion of the brain. Medulla neurons play roles in motion detection, through input from the R1–R6 photoreceptors via the lamina, and in the perception of color, via direct input from the R7 and R8 photoreceptors (Morante and Desplan, 2008). The 40,000 medulla neurons originate from a pseudostratified neuroepithelium (Egger et al., 2011; Sato et al., 2013). During early development, symmetric division expands the stem cell pool. As development progresses, the medial edge of the neuroepithelium is progressively converted into asymmetrically dividing neuroblasts (Figure 1A) (Egger et al., 2010; Yasugi et al., 2010). Medulla neuroblasts sequentially express a series of transcription factors that specify the differentiation of the medulla neurons (Li et al., 2013; Suzuki et al., 2013).

The transition from neuroepithelial cells into neuroblasts occurs in a highly ordered, sequential manner in response to expression of the proneural gene, *lethal of scute* (*l'sc*) (Figure 1B) (Yasugi et al., 2008). Expression of L'sc marks a two- to three-cell-wide boundary between the neuroepithelial cells and the neuroblasts, the so-called transition zone, which moves medially across the neuroepithelium, forming a proneural wave. Within the transition zone, L'sc transiently suppresses Notch activity, triggering the switch from the symmetric, proliferative division of neuroepithelial cells to the asymmetric, differentiative division of neuroblasts (Egger et al., 2010; Yasugi et al., 2010). Progress of the wave is regulated by the orchestrated action of the Notch, epidermal growth factor receptor (EGFR), Fat-Hippo, and JAK/STAT signaling pathways (Egger et al., 2010; Ngo et al., 2010; Reddy et al., 2010; Wang et al., 2011; Yasugi et al., 2010).

We present evidence here that the transition from neuroepithelial cells to neuroblasts in the developing optic lobe is buffered by the microRNA *miR-7. miR-7* is expressed at the transition zone in response to epidermal growth factor (EGF) signaling and is sufficient to promote transition. *miR-7* acts via repression of downstream Notch effectors to limit Notch signaling and promote timely transition. In the absence of *miR-7*, proneural wave progression is disrupted. This disruption becomes more severe under conditions of temperature stress, suggesting that the role of *miR-7* is to act as a buffer to ensure the timely and precise transition from neuroepithelial cells to neuroblasts in the developing optic lobe.

RESULTS

mir-7 Is Expressed at the Transition Zone in the Optic Lobe

Previously we used Targeted DamID to profile gene expression in neuroepithelial cells and neuroblasts of the developing larval optic lobe (Southall et al., 2013). A comparison of the genes that were highly enriched in either neuroepithelial cells or neuroblasts identified genes that may play roles in regulating the transition from neuroepithelium to neuroblasts. We found that *hnRNP-K/miR-7* expression was enriched in neuroepithelial cells (Figure 1C).

The highly conserved microRNA *miR-7* is produced from the primary transcript of *heterogeneous nuclear ribonucleoprotein K* (*hnRNP-K*) in both *Drosophila* (Li and Carthew, 2005) and humans (Choudhury et al., 2013). *miR-7* is predicted to target multiple members of the Notch pathway. The ability of *miR-7* to regulate Notch signaling was of interest, as Notch signaling is known to be essential to maintain neuroepithelial cells, and

This is an open access article under the CC BY license (http://creativecommons.org/licenses/by/4.0/).

OPEN
ACCESS
CellPress

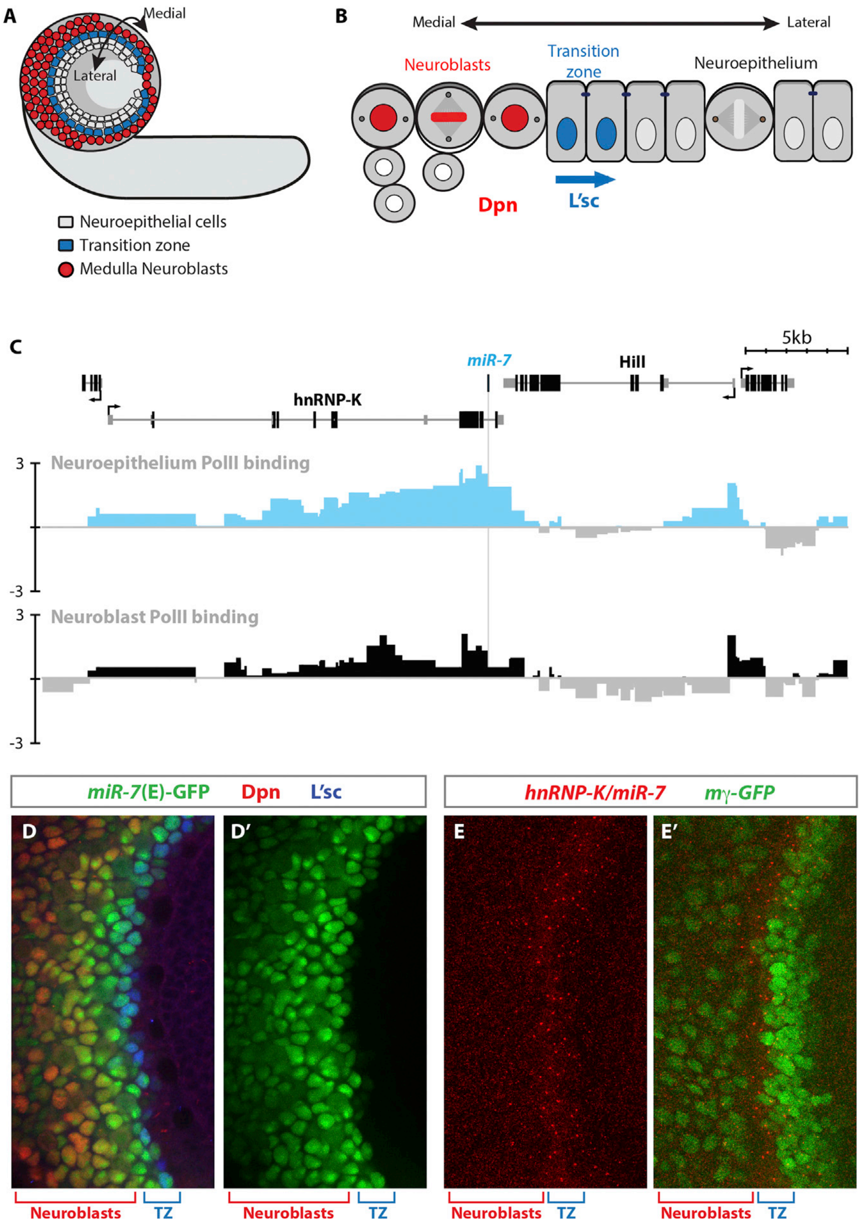

Figure 1. miR-7 Is Expressed in the Transition Zone of the Developing Optic Lobe

(A) Cartoon of a lateral view of the larval brain. Medulla neuroblasts (red) are produced at the lateral edge of the neuroepithelium (gray) following passage through the transition zone marked by the expression of L'sc (blue).

(B) A zoom-in on the transition zone. Symmetrically dividing neuroepithelial cells (gray) are transformed into asymmetrically dividing neuroblasts (marked by expression of Dpn; red) by the lateral movement of the proneural wave. The transition zone is marked by the expression of L'sc (blue).

(C) Differential polymerase (Pol) II occupancy in neuroepithelial cells and neuroblasts presented at GATC fragment resolution (genome release 5.9) shows an enrichment of *HnRNP-K/miR-7* in neuroepithelial cells. Scale bars represent $\log_2$ Dam-Pol II/Dam ratio change.

(D) Expression of miR-7(E)-GFP overlaps with L'sc staining in the transition zone.

(E) RNA FISH against *HnRNP-K/miR-7* shows increased expression in the transition zone (marked by the absence of m-gamma-GFP).

downregulation of Notch activity is required for transition to neuroblasts (Egger et al., 2010; Ngo et al., 2010; Orihara-Ono et al., 2011; Wang et al., 2011; Yasugi et al., 2010). We hypothesized that *miR-7* plays a role in regulating Notch activity at the transition zone.

To investigate *miR-7* function at the transition zone, we determined the precise expression pattern of *hnRNP-K/miR-7* in the neuroepithelium. Examination of the *miR-7* enhancer driving GFP ((*miR-7*)E-GFP) (Li et al., 2009), revealed expression in the transition zone that overlapped precisely L'sc expression (Figures 1D and 1D′), suggesting that *miR-7* is upregulated in the transition zone. We confirmed this expression pattern using single-molecule fluorescence in situ hybridization (FISH) (smFISH) against the *hnRNP-K/miR-7* transcript (Figures 1E and 1E′). We observed a band of increased *hnRNP-K/miR-7* transcription that overlaps the transition zone, marked by the downregulation of E(spl)m-gamma-GFP (Almeida and Bray, 2005; Egger et al., 2011). Similar to the expression of (*miR-7*)E-GFP in the eye imaginal disc (Li et al., 2009), expression of *hnRNP-K/miR-7* in the optic lobe was positively regulated by EGF signaling (Figure S1). This highly specific upregulation of *hnRNP-K/miR-7* transcript at the transition zone suggested a role in regulation of the transition of neuroepithelial cells to neuroblasts.

miR-7 Is Sufficient to Promote the Transition from Neuroepithelial Cells to Neuroblasts and Is Necessary for Robust Transition

To investigate the role of *miR-7* at the transition zone, we generated a UAS-*miR-7* construct and drove its expression in the neuroepithelium using c855a-GAL4 (Manseau et al., 1997). Misexpression of *miR-7* in neuroepithelial cells resulted in disruption of the neuroepithelium and ectopic neuroblast formation (Figures 2A and 2B), similar to what was seen when Notch activity was downregulated throughout the neuroepithelium (Egger et al., 2010). To assess more precisely the effects of *miR-7* misexpression, we generated clones of cells expressing *miR-7*. Clones that spanned the transition zone showed premature neuroblast formation (Figures 2C–2C″). Therefore, *miR-7* is sufficient to convert neuroepithelial cells into neuroblasts.

The available *miR-7* allele is a 6.8-kb deletion generated by P-element excision that partially deletes both *hnRNP-K*

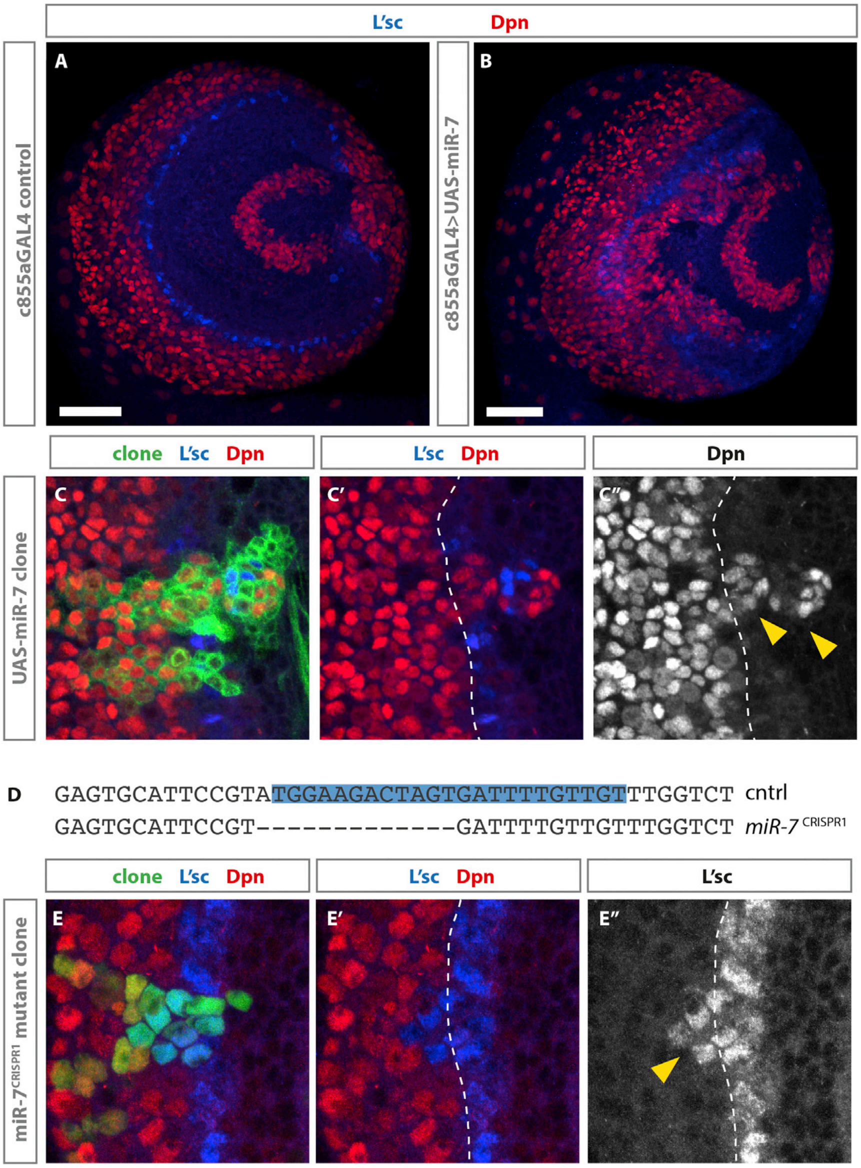

Figure 2. miR-7 Is Sufficient to Promote Premature Transition and Is Necessary for Robust and Timely Transition

(A) Wild-type optic lobe showing expression of L'sc (blue) and Dpn (red).

(B) Expression of UAS-*miR-7* in the neuroepithelium using c855a-GAL4 results in ectopic neuroblast formation (red) throughout the neuroepithelium (compare to A). The scale bar represents 40 μm.

(C–C″) Clonal expression of UAS-*miR-7* results in a pronounced shift in the proneural wave and premature transition from neuroepithelial cells into neuroblasts.

(D) CRISPR of the miR-7 locus generated a 13-bp deletion that removes the 5′ end of *miR-7* named *miR-7*^{CRISPR1}.

(E) MARCM mutant clones of miR-7^{CRISPR1} show a delay in transition and an autonomous increase in the width of the l'sc-positive cells (30%, n = 36; compared to 2.85%, n = 35 in control clones).

and the downstream gene *Hillarian* (Li and Carthew, 2005). To investigate the effect of loss of *miR-7* without disrupting *hnRNP-K* or *Hillarian*, we generated a new *miR-7* CRISPR allele (Figure S2). *miR-7*^{CRISPR1} is a 13-bp deletion that removes the 5′ 12 nt of the 23-nt *miR-7* (Figure 2D) without disrupting neighboring genes. miR-7^{CRISPR1} homozygous mutants were both viable and fertile. Interestingly, homozygous mutants did not display defects in wing development that had previously been attributed to loss of *miR-7* function (Aparicio et al., 2014). Therefore, it is likely that the reduced wing size observed was due to the disruption of *hnRNP-K*, which has been reported to display reduced cell division and increased apoptosis in imaginal discs, resulting in small adult appendages (Charroux et al., 1999). These results highlight the necessity of generating small targeted deletions when investigating the biological functions of microRNAs and, in particular, the advantage of using CRISPR for miRNA mutation.

To assess whether *miR-7* was necessary for the neuroepithelial-to-neuroblast transition, we generated *miR-7*^{CRISPR1} mutant clones that cross the transition zone (Figures 2E–2E″). 30% of mutant clones showed a delay in transition and an autonomous increase in the width of the band of L'sc-positive cells (n = 36). In control clones, only 2.85% (n = 35) showed any cell-autonomous effects on transition. Interestingly, clones that expressed a constitutively active form of Notch also displayed a delay in the onset of neuroblast formation and prolonged expression of L'sc (Yasugi et al., 2010). These results show that *miR-7* plays a role in regulating the timing of the proneural wave and suggest that it may act by negatively regulating Notch signaling.

miR-7 Targets E(spl)m-gamma at the Transition Zone

Predicted *miR-7* binding sites can be found in the 3′ UTRs of many genes encoding downstream effectors and modulators of Notch pathway activity, including members of the Enhancer of split complex (E[spl]-C) and Bearded complex (Brd-C) (Kheradpour et al., 2007; Lai et al., 2005; Robins et al., 2005; Ruby et al., 2007). One of these, E(spl)m-gamma, is known to be expressed in the neuroepithelium (Egger et al., 2011), intriguingly, in a pattern reciprocal to that of *hnRNP-K*/*miR-7* expression (Figure 1E′). To examine whether *miR-7* targets E(spl)m-gamma in vivo, we took advantage of the E(spl)m-gamma-GFP reporter, a genomic fragment containing GFP cloned in frame, 19 amino acids (aas) from the end of E(spl)m-gamma

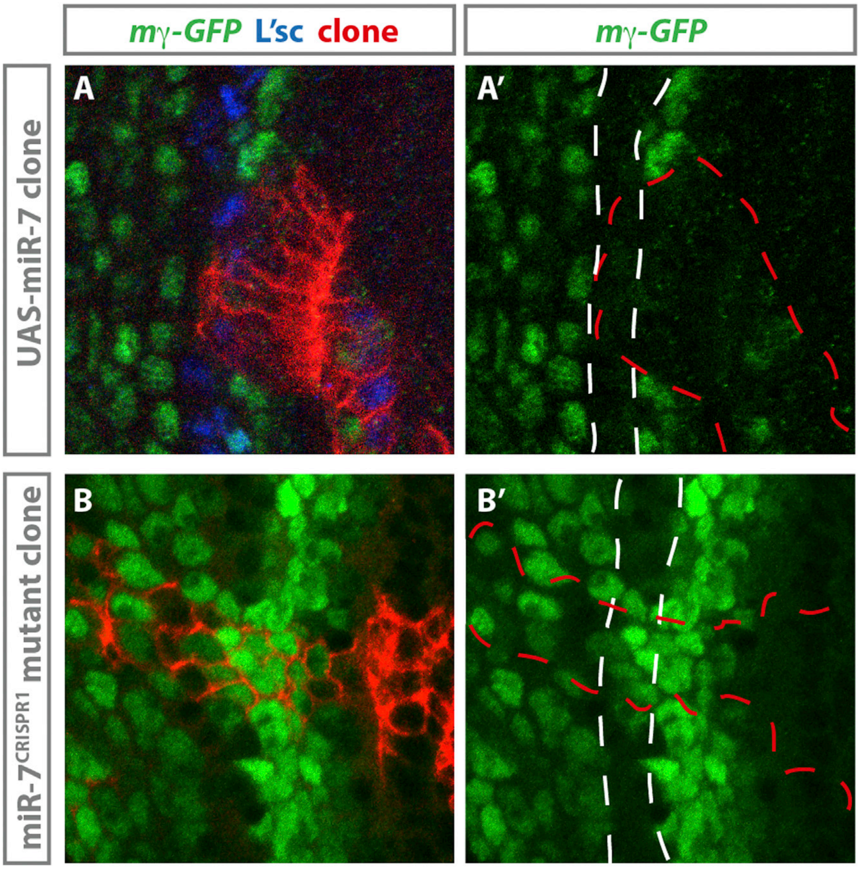

Figure 3. miR-7 Targets Members of the E(spl) Complex at the Transition Zone

(A and A') In (A), clonal expression of UAS-*miR-7* marked by UAS-myr-tdTomato (red) is able to downregulate E(spl)m-gamma-GFP. (A') The transition zone is marked by the white dashed lines; the clone is outlined by the red dashed line.

(B and B') Loss of *miR-7* in a *miR-7*^{CRISPR1} mutant clone marked by UAS-myr-tdTomato (red) results in misregulation of E(spl)m-gamma-GFP expression. (B') The white dashed lines indicate the normal transition zone; the clone is outlined by the red dashed line.

(Almeida and Bray, 2005). Expression of UAS-*miR-7* in clones was able to autonomously downregulate E(spl)m-gamma-GFP at the transition zone (Figures 3A and 3A′). Loss of *miR-7* in *miR-7*^{CRISPR1} mutant clones resulted in a disruption of the normal E(spl)m-gamma-GFP pattern in a subset of clones, consistent with the observed delay in transition (Figures 3B and 3B′). Together, these results confirm that E(spl)m-gamma is a target of *miR-7* at the transition zone and that *miR-7* represses a downstream effector of Notch signaling at the transition zone.

mir-7 Buffers the Neuroepithelial-Cell-to-Neuroblast Transition

Although expression of *miR-7* was sufficient to promote the neuroepithelial-to-neuroblast transition, we only observed a delay in transition in 30% of *miR-7*^{CRISPR1} mutant clones (Figures 2E–2E″), suggesting that transition can occur correctly in the absence of *miR-7* and that only under certain circumstances does the loss of *miR-7* affect timely neuroblast production. This raised the possibility that *miR-7* could be acting as a biological buffer in the developing optic lobe, sufficient to promote transition but necessary only under conditions of physiological stress. *miR-7* had been identified previously as a buffer in photoreceptor and proprioceptor determination (Li and Carthew, 2005; Li et al., 2009). To test whether *miR-7* buffers the neuroepithelial-to-neuroblast transition, we subjected control and *miR-7*^{CRISPR1} mutant larvae to temperature stress, shifting developing larvae between 18°C and 31°C every 2 hr for 2 days. Under normal laboratory conditions, the absence of *miR-7* resulted in the sporadic disruption of the progression of the proneural wave. L'sc-positive cells, which are normally restricted to the transition zone, could be observed trailing behind the wave, either still adjacent to the wave (interpreted as a weak phenotype) or completely isolated from the wave (interpreted as a strong phenotype) (Figures 4B and 4C). Temperature stress increased the severity of the disruption of proneural wave progression in *miR-7*^{CRISPR1} mutant brains ($p = 0.049$, Fisher's exact test) but had no effect on control brains ($p = 1$, Fisher's exact test) (Figures 4A–4C). This increase in severity of phenotype observed under temperature stress demonstrates that *miR-7* buffers the neuroepithelial-cell-to-neuroblast transition.

mir-7 Regulates Notch Effectors to Ensure Timely Transition

We have shown that *miR-7* is able to repress E(spl)m-gamma at the transition zone (Figure 3). *miR-7* is predicted to target a number of other basic-helix-loop-helix (bHLH) transcription factors and Brd genes of the E(spl) complex and Brd complex (Kheradpour et al., 2007; Lai et al., 2005; Ruby et al., 2007). To determine which predicted targets *miR-7* regulates at the transition zone, we performed genetic suppression experiments. We reasoned that, in the absence of *miR-7*, the levels of its target gene(s) should increase and that this increase could be suppressed by reducing the gene dose of the target gene(s). We examined proneural wave progression in the absence of *miR-7* in a background heterozygous for deficiencies that delete large parts of either the E(spl) or the Brd complex, each removing several predicted *miR-7* targets (Figure 4D) (Chanet et al., 2009). While loss of one copy of Df(3) Brd-C1 did not change the severity of proneural wave disruption relative to loss of *miR-7* alone ($p = 0.50$, Fisher's exact test) (Figures 4C and 4H), loss of one copy of the Df(3)E(spl)

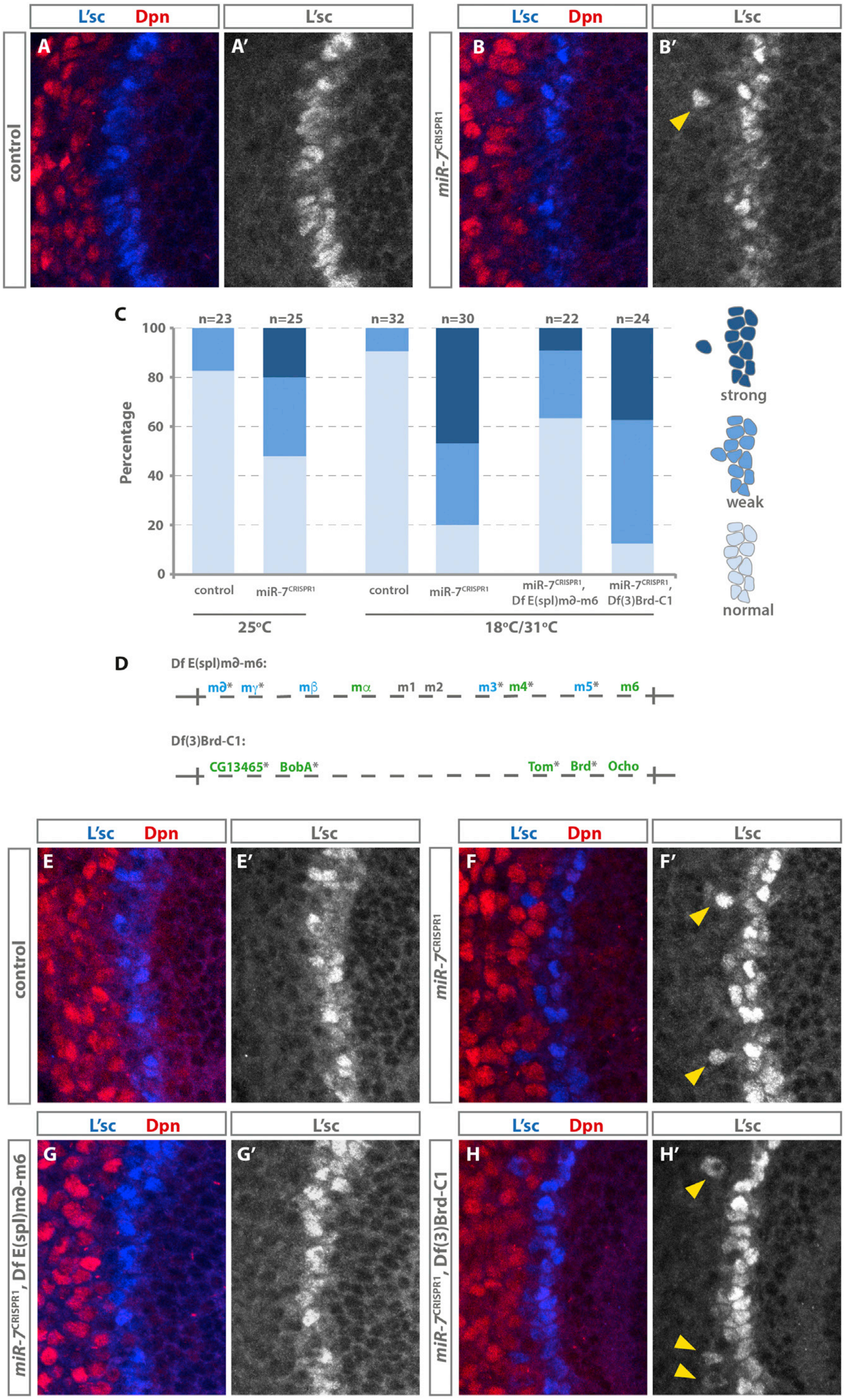

Figure 4. *miR-7* Buffers Transition through Regulation of the E(spl) Complex

(A) The transition zone labeled with L'sc in a control (w1118; +; +) third-instar larval optic lobe.

(B) A miR-7^{CRISPR1} mutant optic lobe showing a defect in the movement of the proneural wave. The yellow arrow indicates a persistence of L'sc staining (blue) outside of the transition zone and a corresponding delay in Dpn expression (red).

(C) Quantification of the transition zone defect. Strong indicates L'sc-positive cells were observed clearly separated from the L'sc cells of the transition zone; weak indicates that L'sc-positive cells were observed trailing but still in contact with the L'sc cells of the transition zone; normal indicates that no extra L'sc-positive cells were observed.

(D) Schematic of the Enhancer of Split complex and Bearded complex deficiencies. bHLH genes are shown in blue; Brd genes are shown in green. Predicted targets of *miR-7* are indicated with an asterisk. CG13465 may correspond to BobB or BobC (http://flybase.org/reports/FBrf0132135.html).

(E–H) Optic lobes from larvae subjected to 2 days of temperature stress, shifting between 31°C and 18°C every 2 hr. Brains are from (E) control (w1118; +; +); (F) miR-7^{CRISPR1}; (G) miR-7^{CRISPR1}, Df E(spl)m-delta-m6; and (H) miR-7^{CRISPR1}, Df(3) Brd-C1. Yellow arrowheads show L'sc-positive cells that persist behind the proneural wave in (F') and (H'). The defect in (B) is suppressed by loss of one copy of Df E(spl)m-delta-m6, as shown in (G), but is not rescued by loss of one copy of Df(3) Brd-C1, as shown in (H).

delta-6 was able to suppress the proneural wave disruption observed in *miR-7*^{CRISPR1} mutant brains under temperature stress ($p = 0.002$, Fisher's exact test) (Figures 4C and 4G). This shows that deregulation of targets within the E(spl)-complex is responsible for the observed defect in proneural wave co-ordination seen in *miR-7*^{CRISPR1} mutants. Therefore, regulation of members of the E(spl) complex by *miR-7* is necessary for buffering the transition from neuroepithelial cells to neuroblasts against environmental stress.

DISCUSSION

We have shown here that *miR-7* targets members of the E(spl) family of bHLH transcription factors to define the boundaries of the transition zone, buffering the transition from neuroepithelial cell to neuroblast in the developing optic lobe. Downregulation of Notch signaling is essential for the neuroepithelial-to-neuroblast transition (Egger et al., 2010; Yasugi et al., 2010). L'sc provides one mechanism for Notch downregulation (Egger et al., 2010). miR-7, expressed in a pattern similar to that of l'sc at the transition zone, provides another, emphasizing the importance of Notch regulation at the transition zone.

The transition zone of the proneural wave mediates the specification of the neuroblasts of the medulla, the largest ganglion of the adult *Drosophila* visual system (Yasugi et al., 2008). The medulla receives information directly from the R7 and R8

photoreceptors of the ommatidia. Similar to the specification of neuroblasts by the proneural wave in the optic lobe, photoreceptor differentiation is triggered by the movement of the morphogenetic furrow across the epithelium of the eye imaginal disc (Sato et al., 2013). As the furrow passes, expression of the proneural gene *atonal* (*ato*) is induced in a stripe that is later refined to the R8 cells by Notch-mediated lateral inhibition (Jarman et al., 1994).

Similar to our observations in the optic lobe, *miR-7* has been shown to play a role in photoreceptor differentiation (Li et al., 2009). Misexpression of *miR-7* results in an increase in Ato expression and R8 cell specification, while a loss of *miR-7* results in a decrease in Ato expression under conditions of temperature stress. These results show that *miR-7* acts to buffer the development of both the medulla and the eye, two tissues that will directly communicate in the adult brain.

miR-7 has been shown to target anterior open (aop; also known as yan) in the eye imaginal disc (Li and Carthew, 2005). Within the neuroepithelium, aop activity helps to repress the neuroepithelial-to-neuroblast transition (Wang et al., 2011). This raises the possibility that *miR-7* targeting of aop could also contribute to its function in buffering the neuroepithelial-to-neuroblast transition and that aop could represent a common target during the progression of the proneural wave and the morphogenetic furrow.

In the adult brain, each ommatidium maps to a columnar unit within the lamina and medulla, providing a retinotopic map of the visual field. Signaling from innervating photoreceptors induces the differentiation of lamina neurons. This direct communication provides a strict control of the mapping of photoreceptor and lamina neuron numbers (Umetsu et al., 2006). In contrast, while final numbers of ommatidia and medulla neurons show some co-ordination based on nutrient availability (Lanet et al., 2013), there is no evidence for direct communication between the eye disc and the developing medulla. The presence of *miR-7* in both the eye imaginal disc and the optic lobe represents an independent but conserved buffer that operates to coordinate appropriate developmental progression in each system, in spite of external environmental fluctuations. The presence of this common buffer provides robustness within each system that may contribute to ensuring the eventual connectivity required for retinotopic mapping of the visual system.

EXPERIMENTAL PROCEDURES

Fly Strains

The following strains were generated: (*miR-7*)E > GFP (Li et al., 2009), *m-gamma-GFP* (Almeida and Bray, 2005), c855a-GAL4 (Manseau et al., 1997), w; Df[E(spl)m-delta-m6 XPd08311-RBe00084]/TM6B (Chanet et al., 2009), w1118; Df(3)Brd-C1/TM6B,Tb (Chanet et al., 2009), UAS-*miR-7* (this study), *miR-7*^{CRISPR1} (this study), and FRT42D *miR-7*^{CRISPR1} (this study). Clones were generated with the MARCM lines: y w hsFLP; FRT40A tub-GAL80/CyO; tub-GAL4/TM6, y w hsFLP tub-GAL4 UAS-GFPnls/FM7; FRT42D tub-GAL80/CyO, y w hsflp^{122}; FRT42D tub-GAL80/CyO; and tub-GAL4 UAS-myr-tdTom/TM6B.

HnRNP-K/miR-7 Stellaris Probes

A set of 48 Stellaris probes was designed against an HnRNP-K-RC transcript and labeled with Quasar 670. Third-instar larval brains were fixed in 4% formaldehyde for 1 hr at room temperature and transferred to 70% ethanol overnight at 4°C. Brains were incubated with 125 μM probes in hybridization buffer (100 mg/mL dextran sulfate, 10% formamide, 2× saline sodium citrate [SSC]) overnight at 45°C and washed in wash buffer (10% formamide, 2× SSC).

Generation of UAS-miR-7

The 88-bp *miR-7* hairpin was amplified using primers forward (fwd): 5′-caagaagagaactctgaatagggaattgggGAGTGCATTCCGTATGGAAG-3′ and reverse (rev) 5′-aagtaaggttccttcacaaagatcctctagAAATGCACGCCGTAAGAAG-3′ and cloned via Gibson assembly into EcoRI and XbaI cut pUAST attB (Bischof et al., 2007). The resulting construct was integrated into attB154.

Generation of miR-7 CRISPR Allele

To generate a *miR-7* CRISPR allele, complementary oligos containing the guide RNA (gRNA) 5′-AAAATCACTAGTCTTCCATA-3′ flanked by BbsI overhangs were annealed and cloned into pCFD3 (Port et al., 2014). This vector was injected into nos-phiC integrase; +; attP2 embryos to generate a stable line expressing the miR-7 gRNA under control of the U6:3 promoter.

Flies expressing the *miR-7* gRNA were crossed to a nos-cas-9 line. Five F1 males were crossed in single-pair crosses to a balancer line, and 10 F2 males were taken from each cross, allowed to fertilize individual females, and then squish prepped and genotyped.

Mutations in miR-7 were detected by sequencing of a 256-bp PCR product, amplified using primers that flank the gRNA cut site. 100% of F1 flies produced offspring with mutations. 58.6% (n = 46) of F2 showed alterations to the *miR-7* locus, generating a total of 20 independent alleles.

Immunohistochemistry

Fixation and immunocytochemistry of larval brains was carried out as described previously (Gold and Brand, 2014). The following primary antibodies and dilutions were used: guinea pig anti-Dpn (1:10,000) and rat anti-L'sc (1:5,000) were generated by C.M. Davidson, E.E.C., and A.H.B. (this study) using constructs that were a kind gift of J. Skeath; chick anti-GFP (1:2,000) and rabbit anti-RFP (1:1,000) from Abcam; and fluorescently conjugated secondary antibodies Alexa 405, Alexa 488, Alexa 546, and Alexa 633 (all 1:200) from Life Technologies.

Statistics

To analyze the effect of temperature shift and genetic backgrounds on the severity of the transition zone defect, we assessed differences in the number of brains exhibiting the strong phenotype via pairwise comparisons (2 × 2 contingency tables) performed with the two-tailed Fisher's exact test.

SUPPLEMENTAL INFORMATION

Supplemental Information includes two figures and can be found with this article online at http://dx.doi.org/10.1016/j.celrep.2017.07.047.

AUTHOR CONTRIBUTIONS

E.E.C. and A.H.B. designed the experiments; E.E.C. carried out the experiments; E.E.C. and A.H.B. analyzed the data and wrote the manuscript.

ACKNOWLEDGMENTS

We thank Richard Carthew and Francois Schweisguth for *Drosophila* lines. We thank Catherine Davidson for help generating antisera, Robert Krautz for help with statistical analysis, and Seth Cheetham for comments on the manuscript. This work was funded by a Wellcome Trust Programme grant (092545), a Wellcome Trust Senior Investigator Award (103792), and a BBSRC Project Grant (BB/L007800/1) to A.H.B. A.H.B. acknowledges core funding to the Gurdon Institute from the Wellcome Trust (092096) and CRUK (C6946/A14492).

Received: December 6, 2016
Revised: May 23, 2017
Accepted: July 18, 2017
Published: August 8, 2017

REFERENCES

Almeida, M.S., and Bray, S.J. (2005). Regulation of post-embryonic neuroblasts by *Drosophila* Grainyhead. Mech. Dev. *122*, 1282–1293.

Aparicio, R., Simoes da Silva, C.J., and Busturia, A. (2014). The microRNA miR-7 contributes to the control of *Drosophila* wing growth. Dev. Dyn. *244*, 21–30.

Bischof, J., Maeda, R.K., Hediger, M., Karch, F., and Basler, K. (2007). An optimized transgenesis system for *Drosophila* using germ-line-specific phiC31 integrases. Proc. Natl. Acad. Sci. USA *104*, 3312–3317.

Chanet, S., Vodovar, N., Mayau, V., and Schweisguth, F. (2009). Genome engineering-based analysis of *Bearded* family genes reveals both functional redundancy and a nonessential function in lateral inhibition in Drosophila. Genetics *182*, 1101–1108.

Charroux, B., Angelats, C., Fasano, L., Kerridge, S., and Vola, C. (1999). The levels of the *bancal* product, a *Drosophila* homologue of vertebrate hnRNP K protein, affect cell proliferation and apoptosis in imaginal disc cells. Mol. Cell. Biol. *19*, 7846–7856.

Choudhury, N.R., de Lima Alves, F., de Andrés-Aguayo, L., Graf, T., Cáceres, J.F., Rappsilber, J., and Michlewski, G. (2013). Tissue-specific control of brain-enriched miR-7 biogenesis. Genes Dev. *27*, 24–38.

Egger, B., Gold, K.S., and Brand, A.H. (2010). Notch regulates the switch from symmetric to asymmetric neural stem cell division in the *Drosophila* optic lobe. Development *137*, 2981–2987.

Egger, B., Gold, K.S., and Brand, A.H. (2011). Regulating the balance between symmetric and asymmetric stem cell division in the developing brain. Fly (Austin) *5*, 237–241.

Gold, K.S., and Brand, A.H. (2014). Optix defines a neuroepithelial compartment in the optic lobe of the *Drosophila* brain. Neural Dev. *9*, 18.

Jarman, A.P., Grell, E.H., Ackerman, L., Jan, L.Y., and Jan, Y.N. (1994). *atonal* is the proneural gene for *Drosophila* photoreceptors. Nature *369*, 398–400.

Kheradpour, P., Stark, A., Roy, S., and Kellis, M. (2007). Reliable prediction of regulator targets using 12 *Drosophila* genomes. Genome Res. *17*, 1919–1931.

Lai, E.C., Tam, B., and Rubin, G.M. (2005). Pervasive regulation of *Drosophila* Notch target genes by GY-box-, Brd-box-, and K-box-class microRNAs. Genes Dev. *19*, 1067–1080.

Lanet, E., Gould, A.P., and Maurange, C. (2013). Protection of neuronal diversity at the expense of neuronal numbers during nutrient restriction in the *Drosophila* visual system. Cell Rep. *3*, 587–594.

Li, X., and Carthew, R.W. (2005). A microRNA mediates EGF receptor signaling and promotes photoreceptor differentiation in the *Drosophila* eye. Cell *123*, 1267–1277.

Li, X., Cassidy, J.J., Reinke, C.A., Fischboeck, S., and Carthew, R.W. (2009). A microRNA imparts robustness against environmental fluctuation during development. Cell *137*, 273–282.

Li, X., Erclik, T., Bertet, C., Chen, Z., Voutev, R., Venkatesh, S., Morante, J., Celik, A., and Desplan, C. (2013). Temporal patterning of *Drosophila* medulla neuroblasts controls neural fates. Nature *498*, 456–462.

Manseau, L., Baradaran, A., Brower, D., Budhu, A., Elefant, F., Phan, H., Philp, A.V., Yang, M., Glover, D., Kaiser, K., et al. (1997). GAL4 enhancer traps expressed in the embryo, larval brain, imaginal discs, and ovary of Drosophila. Dev. Dyn. *209*, 310–322.

Morante, J., and Desplan, C. (2008). The color-vision circuit in the medulla of *Drosophila*. Curr. Biol. *18*, 553–565.

Ngo, K.T., Wang, J., Junker, M., Kriz, S., Vo, G., Asem, B., Olson, J.M., Banerjee, U., and Hartenstein, V. (2010). Concomitant requirement for Notch and Jak/Stat signaling during neuro-epithelial differentiation in the *Drosophila* optic lobe. Dev. Biol. *346*, 284–295.

Orihara-Ono, M., Toriya, M., Nakao, K., and Okano, H. (2011). Downregulation of Notch mediates the seamless transition of individual *Drosophila* neuroepithelial progenitors into optic medullar neuroblasts during prolonged G1. Dev Biol. *351*, 163–175.

Port, F., Chen, H.-M., Lee, T., and Bullock, S.L. (2014). Optimized CRISPR/Cas tools for efficient germline and somatic genome engineering in *Drosophila*. Proc. Natl. Acad. Sci. USA *111*, E2967–E2976.

Reddy, B.V.V.G., Rauskolb, C., and Irvine, K.D. (2010). Influence of fat-hippo and notch signaling on the proliferation and differentiation of *Drosophila* optic neuroepithelia. Development *137*, 2397–2408.

Robins, H., Li, Y., and Padgett, R.W. (2005). Incorporating structure to predict microRNA targets. Proc. Natl. Acad. Sci. USA *102*, 4006–4009.

Ruby, J.G., Stark, A., Johnston, W.K., Kellis, M., Bartel, D.P., and Lai, E.C. (2007). Evolution, biogenesis, expression, and target predictions of a substantially expanded set of *Drosophila* microRNAs. Genome Res. *17*, 1850–1864.

Sato, M., Suzuki, T., and Nakai, Y. (2013). Waves of differentiation in the fly visual system. Dev. Biol. *380*, 1–11.

Southall, T.D., Gold, K.S., Egger, B., Davidson, C.M., Caygill, E.E., Marshall, O.J., and Brand, A.H. (2013). Cell-type-specific profiling of gene expression and chromatin binding without cell isolation: assaying RNA Pol II occupancy in neural stem cells. Dev. Cell *26*, 101–112.

Suzuki, T., Kaido, M., Takayama, R., and Sato, M. (2013). A temporal mechanism that produces neuronal diversity in the *Drosophila* visual center. Dev. Biol. *380*, 12–24.

Umetsu, D., Murakami, S., Sato, M., and Tabata, T. (2006). The highly ordered assembly of retinal axons and their synaptic partners is regulated by Hedgehog/Single-minded in the *Drosophila* visual system. Development *133*, 791–800.

Wang, W., Liu, W., Wang, Y., Zhou, L., Tang, X., and Luo, H. (2011). Notch signaling regulates neuroepithelial stem cell maintenance and neuroblast formation in *Drosophila* optic lobe development. Dev. Biol. *350*, 414–428.

Yasugi, T., Umetsu, D., Murakami, S., Sato, M., and Tabata, T. (2008). *Drosophila* optic lobe neuroblasts triggered by a wave of proneural gene expression that is negatively regulated by JAK/STAT. Development *135*, 1471–1480.

Yasugi, T., Sugie, A., Umetsu, D., and Tabata, T. (2010). Coordinated sequential action of EGFR and Notch signaling pathways regulates proneural wave progression in the *Drosophila* optic lobe. Development *137*, 3193–3203.

Cell Reports, Volume *20*

Supplemental Information

miR-7 Buffers Differentiation in the Developing *Drosophila* Visual System

Elizabeth E. Caygill and Andrea H. Brand

Supplementary Figure 1

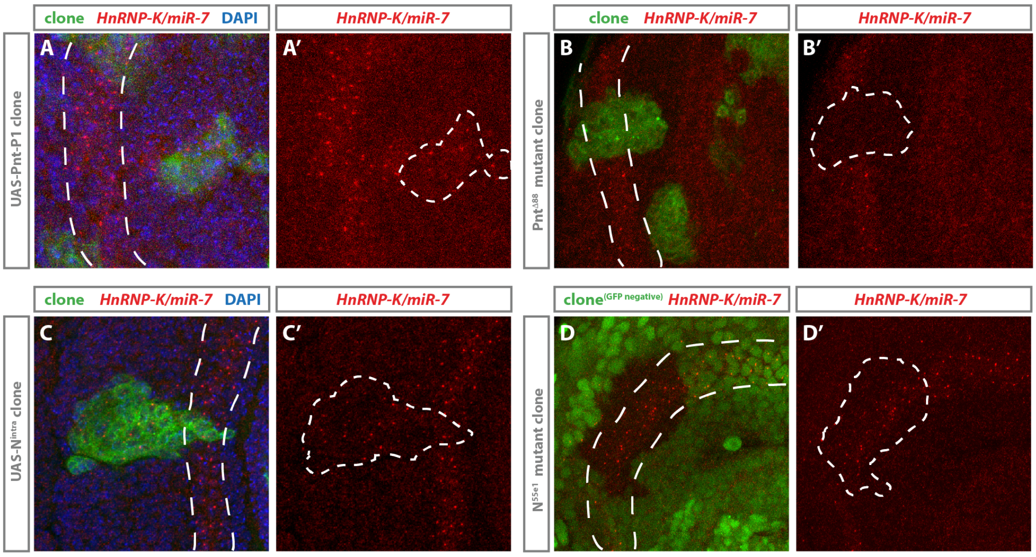

***Supplemental Figure 1. hnRNP-K/miR-7* is regulated by EGF signaling at the transition zone, (Related to Figure 1).**

We tested the ability of *HnRNP-K/miR-7* transcription to respond to alterations in the two major signaling pathways that modulate the transition zone, EGF and Notch. Upregulation of EGF signaling via clonal expression of UAS-Pnt-P1 induces upregulation of *HnRNP-K/miR-7* autonomously and non-autonomously (A). Clonal loss of EGF signaling results in a loss of *HnRNP-K/miR-7* expression (B) suggesting EGF is necessary for expression of *HnRNP-K/miR-7*. Upregulation of Notch signaling via clonal expression of UAS-N^{intra} induces upregulation of *HnRNP-K/miR-7* within the clone (C). However as upregulation of Notch signaling is known to induce EGF activity we tested the effect of loss of Notch signaling and saw that clonal loss of Notch signaling does not result in a loss of *HnRNP-K/miR-7* expression (D) suggesting Notch does not directly regulate expression of *HnRNP-K/miR-7.*

Supplementary Figure 2

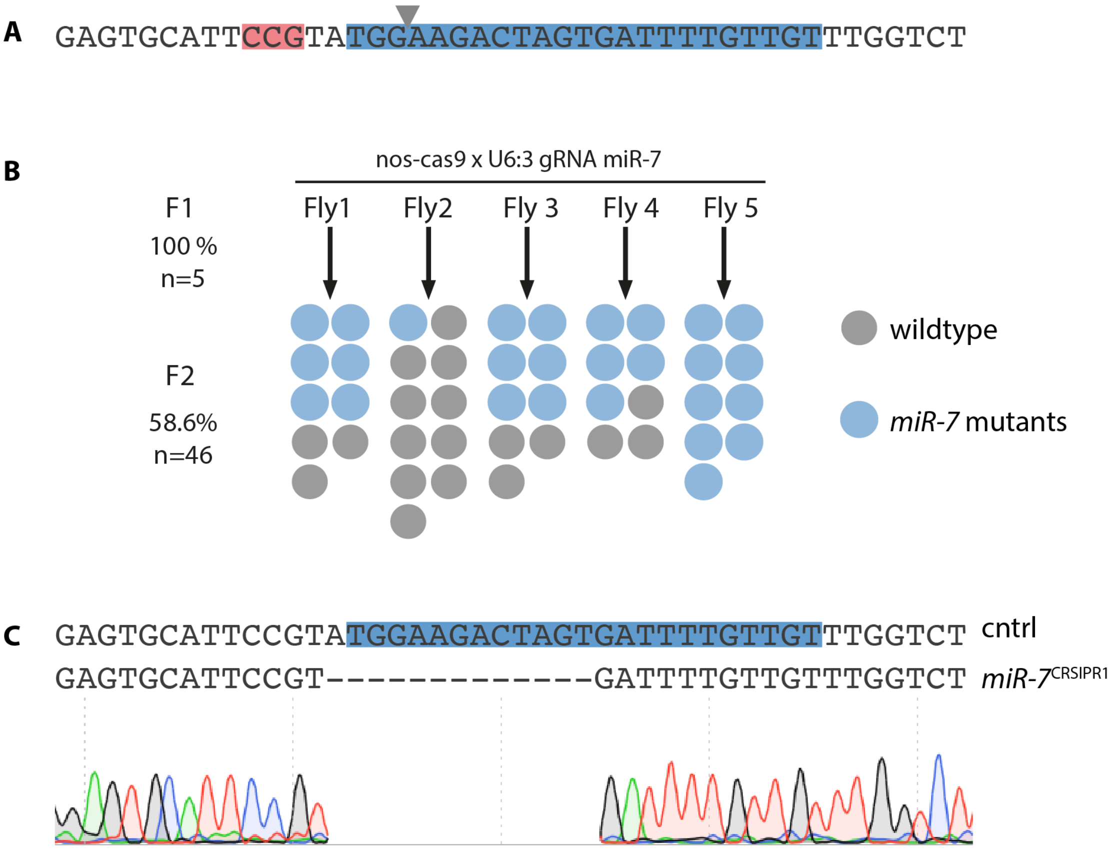

Supplemental Figure 2. **Generation of a *miR-7* CRISPR mutant, (Related to Figure 2).**

(A) The *miR-7* sequence (blue) and surrounding DNA showing the cut site (grey triangle) and the PAM sequence (red) of the gRNA chosen for mutation of *miR-7.*

(B) Efficiency of *miR-7* disruption. nos-cas9 flies were crossed to U6:3 miR-7 gRNA flies. Five F1 male progeny were crossed to balancer lines. 10 F2 male progeny were allowed to fertilize balancer females for 2-3 days then removed from the cross vials and used in diagnostic PCRs. 58.6% (n=46) of F2 progeny tested showed disruption of the *miR-7* locus generating 20 independent alleles.

(C) Sequencing of a *miR-7*$^{\text{CRISPR1}}$ homozygous mutant shows a 13bp deletion.
